# Temporal Expression Classification and Normalization From Chinese Narrative Clinical Texts: Pattern Learning Approach

**DOI:** 10.2196/17652

**Published:** 2020-07-27

**Authors:** Xiaoyi Pan, Boyu Chen, Heng Weng, Yongyi Gong, Yingying Qu

**Affiliations:** 1 School of Information Science and Technology Guangdong University of Foreign Studies Guangzhou China; 2 Department of Big Data Research of Medicine The Second Affiliated Hospital of Guangzhou University of Chinese Medicine Guangzhou China; 3 School of Business Guangdong University of Foreign Studies Guangzhou China

**Keywords:** Temporal expression extraction, Temporal expression normalization, Machine learning, Heuristic rule, Pattern learning, Clinical text

## Abstract

**Background:**

Temporal information frequently exists in the representation of the disease progress, prescription, medication, surgery progress, or discharge summary in narrative clinical text. The accurate extraction and normalization of temporal expressions can positively boost the analysis and understanding of narrative clinical texts to promote clinical research and practice.

**Objective:**

The goal of the study was to propose a novel approach for extracting and normalizing temporal expressions from Chinese narrative clinical text.

**Methods:**

TNorm, a rule-based and pattern learning-based approach, has been developed for automatic temporal expression extraction and normalization from unstructured Chinese clinical text data. TNorm consists of three stages: extraction, classification, and normalization. It applies a set of heuristic rules and automatically generated patterns for temporal expression identification and extraction of clinical texts. Then, it collects the features of extracted temporal expressions for temporal type prediction and classification by using machine learning algorithms. Finally, the features are combined with the rule-based and a pattern learning-based approach to normalize the extracted temporal expressions.

**Results:**

The evaluation dataset is a set of narrative clinical texts in Chinese containing 1459 discharge summaries of a domestic Grade A Class 3 hospital. The results show that TNorm, combined with temporal expressions extraction and temporal types prediction, achieves a precision of 0.8491, a recall of 0.8328, and a F1 score of 0.8409 in temporal expressions normalization.

**Conclusions:**

This study illustrates an automatic approach, TNorm, that extracts and normalizes temporal expression from Chinese narrative clinical texts. TNorm was evaluated on the basis of discharge summary data, and results demonstrate its effectiveness on temporal expression normalization.

## Introduction

Temporal information, expressions of words or phrases about time, is vital to process and understand data related to time dimension [1]. The automatic extraction of temporal information using natural language processing (NLP) techniques has become a research hotspot [2]. The extraction and summary of events that contain temporal information in chronological order play a key role in many NLP applications such as text summarization [3]. In practice, temporal information can be applied to many tasks (eg, temporal indexing for indicating medical entities of a united timeline to help comprehend clinical notes and further analysis) [4].

In the medical domain, temporal information has been proved to be useful in clinical research advances and remains essential to the analysis and understanding of clinical events hidden in narrative clinical texts [2]. As typical medical data sources, electronic medical records (EMRs) are collections of electronically stored records that keep medical treatment information of patients in the hospital. These EMRs contain massive unstructured narrative clinical texts (eg, discharge summaries, progress notes). Almost all types of records contain temporal expressions (TEs) as an important indication of clinical information about disease treatment [5,6]. Thus, extraction and normalization of temporal information from these unstructured texts is exceedingly valuable for clinical timeline construction as well as diagnosis procedure identification for clinical analysis [7].

However, the extraction and normalization of clinical temporal information presents difficulties in the current situation. Narrative clinical texts authored by clinical researchers normally includes a large amount of domain terminologies, making them relatively more complicated than other types of narrative texts [8]. Additionally, certain TEs written in different recording habits also increase difficulty of extraction from texts [9]. Most available systems for temporal information processing from EMRs are designed for English texts, while just a small number of them are proposed for Chinese texts [10]. In addition, most shared tasks (eg, the Informatics for Integrating Biology and the Bedside [i2b2] NLP Challenge and several Clinical TempEval Tasks) concentrate on TE extraction and relation identification and seldom pay attention to TE normalization. Relatively speaking, there are several challenges in the task of TE normalization. For instance, TEs are expressed in various formats, causing difficulties in normalization [7]. In addition, certain TEs are dependent on each other, and the normalization needs to identify and compute their reference time. Therefore, time resolution is required to determine whether a TE needs a reference time and how to identify an appropriate reference time for normalization correctly.

Since 2006, multiple clinical NLP shared tasks have been released for open participation, which delivered positive impacts on the development of clinical NLP research [11]. Temporal information processing (including temporal information extraction, temporal relation identification, and TE normalization) was involved in these tasks. In 2012, the sixth i2b2 NLP Challenge concentrated mainly on temporal relation extraction from clinical narratives [12]. A corpus of clinical discharge summaries containing annotated events and TEs was provided in this challenge. The task comprised extraction of (1) clinical events containing medical concepts (eg, clinical departments, tests) and events associated with the clinical timeline of patients including admissions, transfers among different departments, and so on; (2) TEs, including the types of date, time, duration, or frequency (the standardized value of extracted TEs must refer to an International Organization for Standardization [ISO] specification standard); and (3) temporal relations between TEs and clinical events [12]. Other similar tasks were Clinical TempEval 2015 Task 6 [13] and Clinical TempEval 2016 Task 12 [14]. In 2017, SemEval-2017 Task 12 [15] focused on timeline extraction in the clinical domain. This task used pathology reports as well as clinical notes of cancer patients as experiment data and proposed a domain adaptation problem in temporal information processing. Data from colon cancer patients was selected as the training dataset, and data from brain cancer patients was used as the testing dataset. In the task, MacAvaney et al [16] presented a supervised learning approach for TE extraction and event spans, including conditional random fields (CRFs) and decision tree ensembles.

There are clinical EMRs in different languages and a good amount of research on processing temporal information of EMRs. However, most of the research and systems are designed for English TE extraction and normalization. Luo et al [17] developed a method based on CRF for extracting temporal constraints from eligibility criteria in clinical studies. Chang et al [18] applied a method combining regular expression rules, compositional rules, and filtering rules to identify TEs in text. For the purpose of temporal analysis, Tao et al [19] proposed an ontology-based method for temporal information representation of vaccine adverse events. A comprehensive approach consisting of regular expression, pattern matching, and machine learning was developed by Sohn et al [20] for temporal information processing. Kovačević et al [21] developed a system in which rules were applied to identification and normalization of TEs and a CRF approach was used for events and temporal identification. In order to identify temporal relationship of entities, Chang et al [22] designed a hybrid method containing a rule-based approach and a maximum entropy model. Sun et al [1] transformed the task of normalizing relative and incomplete temporal expressions (RI-TIMEXes) from narrative clinical texts into a multilabel classification problem and developed a normalization system that included an anchor point classifier, anchor relation classifier, and RI-TIMEX text span parser based on rules. Wang et al [23] presented an approach based on shallow syntactic information and crude properties of extracted event and temporal entities for temporal information tagging and relation extraction. Zhu et al [24] proposed an integrated method based on syntactic parsing for extracting structured medical information and associating temporal information from online health communities. In the method, temporal and medical phrase extraction was regarded as a series of tagging, and temporal relation identification was regarded as a classification problem. Lee et al [25] indicated that the main category of temporal relations is direct temporal relation, which contained significant information required for clinical applications. They constructed a corpus composed of direct temporal relations between events and TEs and proposed an automated support vector machine–based system for direct temporal relation. Meanwhile, research related to Chinese TE extraction and normalization was reported. Wu et al [26] proposed a temporal parser to extract and standardize Chinese TEs. Zhou et al [27] established a framework concentrating on processing narrative clinical records in Chinese, including a regular expression matching–based method for TE identification and an approach for temporal relationship extraction using CRF. Li et al [28] further developed Chinese HeidelTime recourses to solve problems in Chinese temporal tagging (extraction and normalization). For the purpose of extracting and normalizing TE from Chinese clinical texts, Liu et al [10] designed a system containing a set of rules for each type of TE. Hao et al [9] presented an approach called temporal expression extractor combining heuristic rules with a pattern learning method for TE extraction and normalization in multilingual narrative clinical texts. In general, existing research on TE extraction and temporal relationship extraction has not achieved enough performance for clinical research practice. Particularly, very little research is concentrated on TE normalization from Chinese narrative clinical texts, and most of it is rule-based strategy only.

In this paper, we proposed a hybrid method named TNorm by incorporating a rule-based and a pattern learning-based strategy for TE extraction, classification, and normalization from Chinese narrative clinical texts. TNorm aimed to solve difficulties caused by various formats of TEs and reference time identification for each TE. In TNorm, two groups of patterns were automatically generated from annotated Chinese clinical discharge summaries. The first group was learned and combined with a set of heuristic rules for extracting TEs. After that, TNorm applied a list of extracted temporal features to classify those expressions into a list of temporal types with the help of machine learning algorithms. Finally, combining with rules, the second group of patterns was generated and applied to normalize the identified and classified TEs. The innovation of TNorm was on the combination of rules and pattern learning to solve the difficulties mentioned for Chinese TEs extraction and normalization. In addition, TNorm is compatible with existing classification algorithms to combine the tasks of temporal extraction and normalization. The TEs extracted and normalized by TNorm could be used to generate a corresponding medical events timeline from Chinese narrative clinical texts.

In order to evaluate the performance of the proposed method, we used 1495 unstructured discharge summaries of breast cancer patients from a 3A hospital in China, among which 900 discharge summaries were randomly selected and manually annotated. In temporal type classification, TNorm with a randomizable filtered classifier (RFC) achieved a macro-average F1 score of 0.9573. In the evaluation of normalization, TNorm achieved a precision of 0.8491, recall of 0.8328, and F1 score of 0.8409. The experiment results demonstrated that TNorm has reliable performance on TE extraction and normalization.

## Methods

### Overall Framework

**Figure 1:**
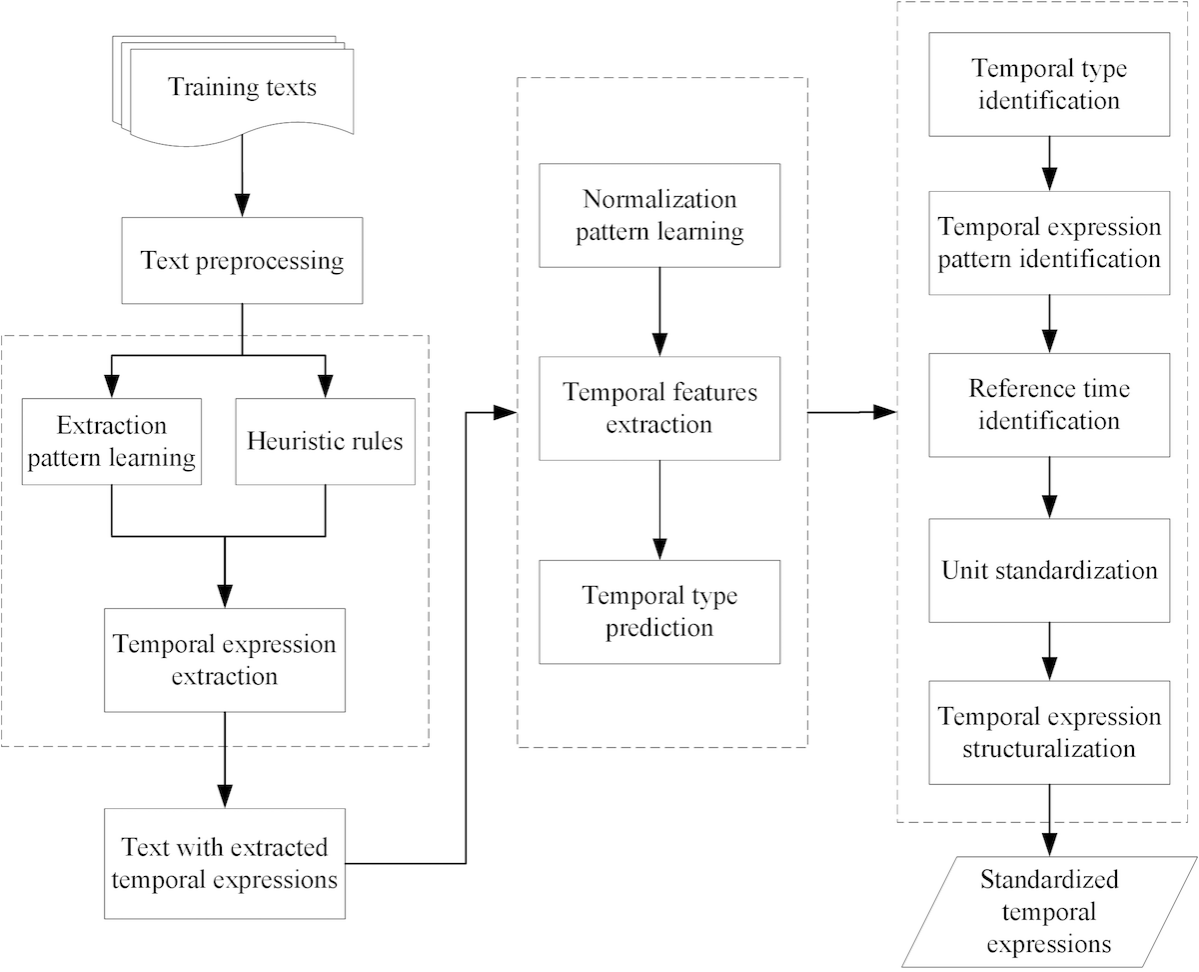
Framework of TNorm.

An automatic method called TNorm was designed for TE extraction and normalization of narrative Chinese clinical texts. It incorporates heuristic rules, automatically learned patterns, and machine learning algorithms and presented a temporal representation as a triple *TE* = <*M*, *A*, *N*>. *TE* denotes a set of temporal mentions as *M*, a set of type attributes of *M* as *A*, and a set of mention values in normalized form as *N*. TNorm transforms TEs into normalized format by referring to two international standards: (1) TimeML [29], a formal specification language for events and TEs, and (2) ISO 8601 [30]. These reference standards are commonly used in many international challenge tasks (eg, 2012 i2b2 Challenge, Clinical TempEval Task). In general, TNorm is proposed to solve the following tasks: (1) extracting temporal mentions *M* from narrative clinical texts, (2) predicting the attributes *A* of mentions *M*, and (3) achieving normalized TE values *N* by standardizing the values of *M*. The framework of TNorm is presented in Figure 1.

In the extraction process, TEs of available texts are extracted in combination with learned pattern and heuristic rules. Then, in the process of temporal type prediction, temporal features are extracted from the texts with annotated TEs to predict temporal types. Finally, the predicted temporal type and another set of learned patterns are combined in the process of normalization.

To extract TEs, we apply a hybrid approach to deal with narrative Chinese EMRs [9] since this paper mainly focuses on mention type classification and expression normalization. The approach analyzes the annotated TE and summarizes a group of temporal features, by which a list of heuristic rules is established. After that, a list of extraction patterns, used to identify TEs, is automatically learned from clinical training datasets. These extraction patterns are then combined with heuristic rules to extract TEs from clinical texts.

### Reference Standard

In the normalization of TEs, two reference standards are adopted: TimeML and ISO 8601. TimeML is applied to define the annotation tags of TEs and ISO 8601 format to standardize the value of TEs.

TimeML includes seven defined tags, <EVENT>, <TIMEX3>, <SIGNAL>, <MAKEINSTANCE>, <TLINK>, <SLINK>, and <ALINK>, which are used to annotate different types of objects. Therefore, we use the tag <TIMEX3> in TNorm to annotate TEs. Accordingly, the attributes defined in <TIMEX3> are divided into nonoptional and optional attributes to represent TEs more accurately. Nonoptional attributes are adopted in TNorm with three indicators, Timex ID number (tid), Type, and Value. In addition, anchorTimeID, an optional attribute for recording the reference TE ID, is used merely when the currently processed TE requires a reference time. For example, a TE is annotated as follows: <TIMEX3 tid=“[ID number]” Type=“[DATE|DURATION|SET|TIME]” Value=“[standardized value of TE]” anchorTimeID=“[reference time ID]”>[TE]</TIMEX3>.

The value attribute is given referring to ISO 8601 format, which defines a widely accepted representation (eg, “YYYY-MM-DD”) for date and time. Based on the representation, ISO 8601 states a series of standardized representation formats. Table 1 shows the formats defined in TNorm.

**Table 1:** International Organization for Standardization 8601 formats defined in TNorm.

Format	Temporal expression in Chinese	Value
YYYY-MM-DD	2014年6月3日	2014-06-03
YYYY-MM	2014年6月	2014-06
YYYY	2014年	2014
YYYY-MM-DDThh:mm:ss	2014年6月3日上午7点20分4秒	2014-06-03T07:20:04
PnYnMnDTnHnMnS	两年五个月	P2Y5M

### Temporal Type Predication

**Figure 2:**
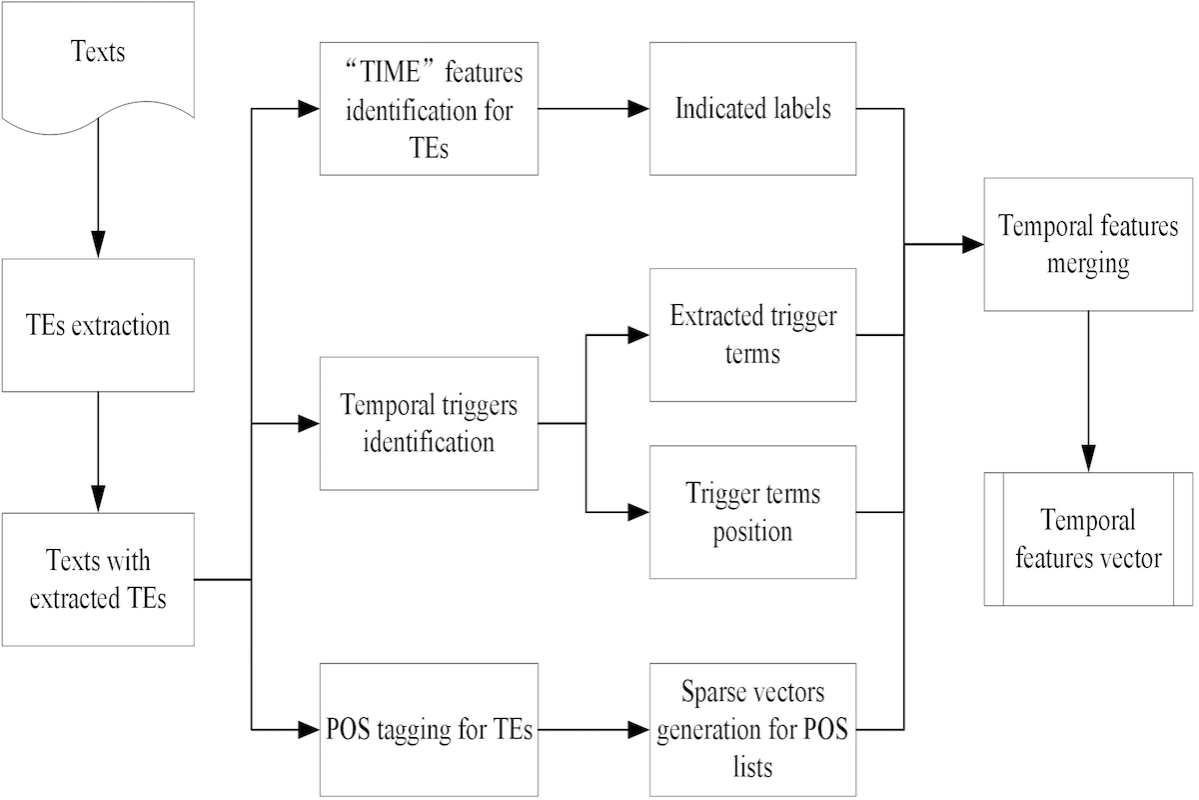
Flow of temporal features extraction.

We identified and extracted a group of temporal features to predict the types of extracted TEs by using machine learning algorithms. We treated the temporal type prediction process as a multiclassification task and used TNorm to leverage machine learning algorithms for prediction. In TNorm, the following temporal features are identified and extracted from TEs in clinical training datasets: (1) part-of-speech tags of TEs, processed and generated by Stanford CoreNLP [31]; (2) trigger terms (eg, temporal units); (3) trigger positions, the relative positions of trigger terms; and (4) indicated labels, labels that indicate whether TEs contain typical features of type “TIME.” The process of extracting these temporal features is presented in Figure 2.

Through the extraction process of TNorm, TEs in clinical texts are extracted and annotated. In the temporal features extraction process, TNorm identifies typical “TIME” features of the extracted TEs for generating corresponding indicated labels. Through identifying temporal triggers from extracted TEs and their context, TNorm extracts temporal trigger terms and their corresponding positions. In addition, TNorm applied the Stanford CoreNLP to generate corresponding part-of-speech tag lists of the extracted TEs and transform the tags into sparse vectors. Finally, the indicated labels, extracted trigger terms, positions of trigger terms, and sparse vectors are merged into new vectors as the temporal feature vectors of TEs. The feature vectors extracted from the training dataset are processed by machine learning algorithms, which are applied to generate a classification model for temporal type prediction. In this paper, we use the Waikato Environment for Knowledge Analysis (Weka), a machine learning toolkit [32], to use classification algorithms. The initial parameters of classification algorithms set by default in Weka are used in TNorm. After that, classification algorithms predict temporal types of extracted TEs on the testing dataset with temporal feature vectors. We classified the TEs into four types according to TimeML: (1) TIME, (2) DATE, (3) SET, and (4) DURATION. TNorm selects proper normalization process for different TEs based on their temporal types and formats. For instance, regular TEs presented as the DURATION or other temporal types are normalized directly (eg, “*一个月*”, “2014/10/11 7:48:16” and “2014-10-13” are normalized as <TIMEX3 tid=“t1” Type=“DURATION” Value=“P1M”>, < TIMEX3 tid=“t2” Type=“TIME” Value=“2014-10-11T07:48:16”>, and <TIMEX3 tid=“t3” Type=“DATE” Value=“2014-10-13”> by TNorm).

### Temporal Expression Normalization

To normalize extracted TEs, a list of normalization patterns is automatically learned and generated. For the pattern generation process, a set of candidate patterns is first extracted from the annotated training dataset and then matched back to original texts in the training dataset for validation. The patterns that have confidence scores higher than a predefined threshold are kept. Finally, heuristic rules are summarized through manual observation and applied to normalize the extracted TEs. The learned patterns are used to validate and correct normalized temporal values simultaneously.

After temporal type prediction, TNorm determines reference time for the extracted TEs on the basis of their TE formats and contexts. The reference time plays a key role in transforming the values of TEs into a standard format, so the identification of suitable reference time is important in the normalization process. We proposed three strategies to identify reference time.

When using occurrence time of critical events, certain occurrence times of clinical-related events in narrative texts can be regarded as the reference time if it is highly relevant to current TEs. Through analyzing the context, we classified some events as critical events according to the distance between their locations and TEs in the same sentences. By analyzing the characters in discharge summaries, we found that critical events in an EMR consist of admission, discharge, operation, chemotherapy, and so on. A group of clinical-related events (eg, “*回院*” [back to the hospital], “*化疗后*” [after chemotherapy], “*化疗后*” [postoperation]) are summarized. TNorm detects these events in unstructured clinical texts to acquire corresponding reference time.

When using reference time of special phrases, certain phrases that have a strong relationship with some critical events can be classified as identifiers. We take the time of a critical event as the reference time of all TEs in corresponding special phrases in the same paragraphs. For example, the “*入院诊断*” (admitting diagnosis) phrase is related with the critical event “*入院*” (admission), and the reference time of TEs in the paragraph is the occurrence time of “*入院”* (admission).

**Figure 3:**
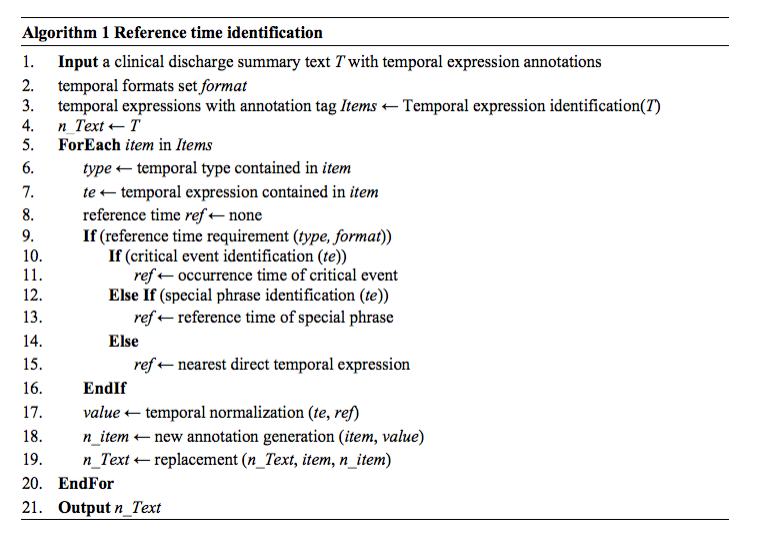
Reference time identification.

When using nearest direct TEs, if the types of TEs are not “DURATION” and they can be normalized directly without reference time, they are defined as direct time that can be applied as the reference time for further normalization. Table 2 shows examples of identifying the reference time of indirect TEs. Figure 3 shows the algorithm for reference time identification.

As shown in Table 2, the critical event of the TE “*第7、10、14天*” is “*化疗*” (chemotherapy). The occurrence time of the event can be extracted from narrative texts and thus the reference time of this TE is the date of chemotherapy (“*化疗*”). For the second example, the TE “*72小时后*” is irrelevant to any critical clinical-related event but is related in a special phrase “*出院医嘱*” (discharge instruction) whose reference time is the date of discharge. In the EMR, the date of discharge exists, and thus the reference time of this TE is the same as that of the special phrase. Nevertheless, if the reference time of the special phrase is not mentioned in the text, such as in the third example, we choose to identify the nearest direct time.

All TEs are normalized after accomplishing the processes mentioned above. However, using heuristic rules alone may result in incorrect normalization results. Some TEs may connect with more than one medical entity (eg, “*主诉:右乳腺癌术后3月余,返院行第7次化疗*” [Chief complaint: More than 3 months after the right breast cancer surgery, patient returned to the hospital for 7th chemotherapy]). The TE is “*3月余*” (more than 3 months) and the medical entities are “*右乳腺癌术后*” (after the right breast cancer surgery) and “*返院*” (returned to the hospital). According to the rule-based method, the reference time of this TE is the occurrence time of “*返院*” (returned to the hospital) and the calculated normalization date is 3 months later than the reference time. However, the correct value of the TE should be equal to the reference time.

**Figure 4:**
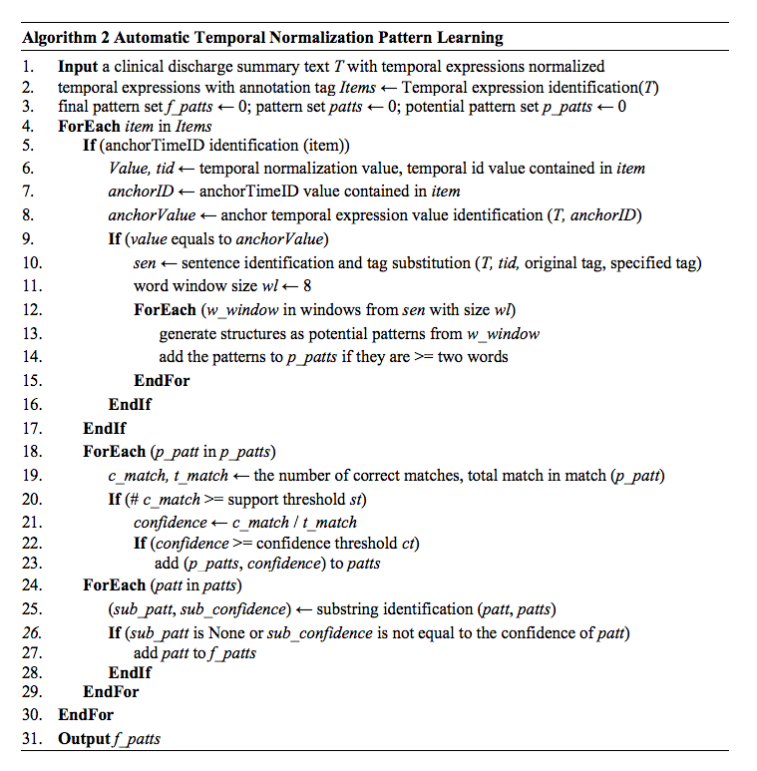
Automatic temporal normalization pattern learning.

To rectify such issues, we applied TNorm to automatically extract a list of patterns from the narrative clinical training dataset with labeled TEs. The detailed procedure of pattern extraction includes the following steps:

1. Label identification: we use the Natural Language Toolkit (NLTK) to split texts into sentences and apply regular expressions to identify TEs that use their reference time as normalization value
2. Temporal label substitution: for retaining contextual information and conveniently extracting patterns, initial TE tags identified in step 1 are substituted by given tags
3. Potential temporal patterns extraction: in the algorithm, given tags and their adjoining words are extracted, and the maximum length of pattern is stipulated. A tag can be contained in several different patterns and all extractive patterns with prescriptive length are regarded as potential patterns
4. Pattern validity verification: the algorithm verifies availability of every potential pattern through applying it to the original dataset and calculates its matching accuracy that can be used as its confidence score
5. Pattern filtration: depending on experiments, the threshold of confidence score is regulated as 0.8. A pattern is adopted merely when its confidence score is higher than the threshold or identical to it. The rest of the patterns are deleted
6. Remove the patterns having substrings: in the filtered pattern group, several patterns are substrings of other patterns. For simplifying the pattern group, a pattern with other substring in the group is removed. Figure 4 shows the algorithm for automatic temporal normalization pattern learning

For instance, when applying the algorithm to an annotated sentence “*遂于<TIMEX3 tid=“t9” Type=“DATE” Value=“2014-09-22” anchorTimeID=“t3”>22/9</TIMEX3>行右乳癌改良根治术*”, the tag and TE are replaced with a given tag (eg, “*遂于<TIMEX3>行右乳癌改良根治术*”). Fifteen patterns are extracted from this sentence, from which the support and confidence scores are calculated. After comparing the threshold and identifying the substring, there are three patterns left, “*<TIMEX3>办*”, “*予<TIMEX3>*”, and “*可予<TIMEX3>*,” which are combined with rules to extract and normalize TEs from texts.

**Table 2:** Examples of temporal expressions and their corresponding reference time in texts.

Example in text	Temporal expression	Critical event	Special phrase	Reference time
*出院医嘱* *: 1* *、化疗后第* *7* *、* *10* *、* *14* *天复查血象;*	*第* *7* *、* *10* *、* *14* *天*	*化疗*	*出院医嘱*	date of chemotherapy
*出院医嘱* *: 1* *、保持伤口清洁干燥* *,72* *小时后自行拆除绷带* *;*	*72* *小时后*	none	*出院医嘱*	date of discharge
*出院情况* *:* *目前患者第一次化疗结束* *,* *未诉特殊不适* *,* *交代相关注意事项后* *,* *准予出院。*	*目前*	none	*出院情况*	nearest direct time

## Results

### Datasets

The experiment dataset contains 1459 Chinese discharge summary texts of patients with breast cancer from a 3A hospital in mainland China. We randomly selected 900 EMRs and manually annotated all TEs. A TE was labeled with tag “<TIMEX3></TIMEX3>” and normalized as the standard form for evaluation. According to the TimeML standard, a label needs to contain four attributes: (1) tid, the index of temporal information in the record; (2) Type, the temporal type; (3) Value, the normalized date of a TE; and (4) anchorTimeID, the index of the reference time of the current TE. Each TE has a unique tag (eg, <TIMEX3 tid=“t1” Type=“TIME” Value=“2014-10-11T07:48:16”>2014/10/11 7:48:16</TIMEX3>, <TIMEX3 tid=“t7” Type=“DATE” Value=“2014-08-02” anchorTimeID=“t2”>1 周前</TIMEX3>, <TIMEX3 tid=“t4” Type=“DURATION” Value=“P1Y”> 1年余</TIMEX3>, <TIMEX3 tid=“t13” Type=“SET” Value=“[10-29]” anchorTimeID=“t11”> 第7、9、14 天</TIMEX3>). These 900 EMRs contain 12,096 TEs (13.44 TEs per text). Details of the training and testing datasets are illustrated in Table 3.

**Table 3:** Statistics of the dataset containing Chinese discharge summary texts.

Datasets	Texts, n	Temporal expressions, n	Temporal expressions per text, mean
Training	450	5966	13.26
Testing	450	6130	13.62
Total	900	12,096	13.44

### Evaluation Metrics

**Figure 5:**
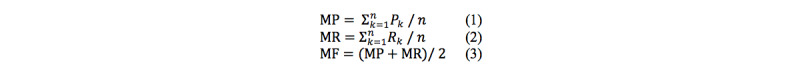
Calculation equations of evaluation metrics macro-average precision, macro-average recall, and macro-average F1-measure. MP: macro-average precision; MR: macro-average recall; MF: macro-average F1-measure.

The task of temporal type prediction can be regarded as a multiclassification task. Since a classification algorithm may display different classification capabilities on different types of TEs and the number of temporal types is rather different in a text, the traditional precision, recall, and F1 are not appropriate to indicate the actual classification capability of a classification algorithm. For instance, a classification algorithm may obtain high precision of “TIME” (one temporal type) prediction but poor precision of “SET” (one temporal type) prediction. It will perform better in a dataset that contains more “TIME” than “SET” and worse in the opposite condition. Therefore, to reduce the differentiation, we applied macro-average precision, macro-average recall, and macro-average F1-measure rather than the traditional precision, recall, and F1 to evaluate the prediction result, as shown in the following equations (Figure 5).

In the equations, *n* represents the quantity of labels while *k* is a number that represents different labels. *P_k_* and *R_k_* stand for the precision and recall, respectively, of the label that corresponds to *k*.

**Figure 6:**
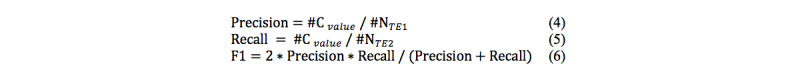
Calculation equations of evaluation metrics precision, recall and F1-measure.

A normalized TE is considered correct only when the extracted expression and its normalized value are completely identical to the manually annotated result. In the comparison of normalization performance, the metrics precision, recall, and F1-measure are used to evaluate the performance of TNorm. Based on the definition, calculations of the metrics are shown in the following equations (Figure 6).

#*C_value_* represents the number of TEs that are correctly extracted and normalized (with correct value of the attribute “Value”), #*N_TE1_* represents the number of TEs that are normalized by TNorm, and #*N_TE2_* represents the number of TEs that should be normalized in the dataset.

### Outcome

For temporal type prediction, TNorm extracted temporal features vectors (as mentioned in the Methods section) from the training dataset and combined machine learning algorithms with these feature vectors. Since the process of temporal type prediction could be treated as the multiclassification tasks, we used Weka to classify temporal types with 10-fold cross-validation. In a classification task, the main influence factors were extracted features and selected classification algorithms. Thus, the performance of a list of algorithms including logistic, decision table, and k-nearest neighbor (KNN) was calculated and ranked by macro-averaged F1-measure (top 10 only). As the result reported in Table 4 shows, RFC achieved the best F1-measure (0.9573). RFC is a variant of a simple filtered classifier that requires either the base learner or the filter to implement a random interface. In this experiment, the base classifier selected in RFC was KNN(k=1) and the filter selected in RFC was random projection, which was used to reduce the dimensionality of vectors. As shown in the table, the KNN algorithm could perform well individually. Through analysis, we determined that the types, relative position, and context of TEs in each discharge summary were mostly similar. Therefore, most initial generated temporal feature vectors with the same temporal types were close in distance. However, the vectors processed by the filter were probably closer in distance with reducing invalid attributes. As a consequence, RFC that combined KNN with random projection in this experiment achieved high performance and was selected as the baseline machine learning algorithm in TNorm, used in the following normalization procedure.

The main parts of the normalization process with TNorm included heuristic rules and pattern learning. For verifying the validity of pattern learning in temporal normalization, the temporal type prediction process and temporal normalization process (as mentioned in Methods) were applied without the application of automatic temporal extraction. We used the same classification algorithm to predict temporal types and compared the effectiveness of the method under two conditions: with rules only and with both rules and the generated patterns. In the TE normalization task, the normalization result of each TE was unique. The normalization result generated by the approach could only be divided into right and wrong. Therefore, the evaluation metric Accuracy was used.

In the metric Accuracy = #Correct / #N_TE_, #Correct represented the number of TEs with correct value of the attributes “Type” and “Value” in the testing dataset and #N_TE_ represented the number of TEs that should be normalized in the testing dataset. The top 5 classification algorithms were respectively combined with the two method models: rule only and rule plus pattern, which were contained in TNorm to normalize TEs. Based on the testing dataset containing 6130 TEs, as the result shows in Table 5, the strategy of method with rules achieved an accuracy of 0.8587, while the second condition with both the rules and pattern learning achieved an accuracy of 0.8654, demonstrating a positive influence of the learned patterns in the normalization process.

**Figure 7:**
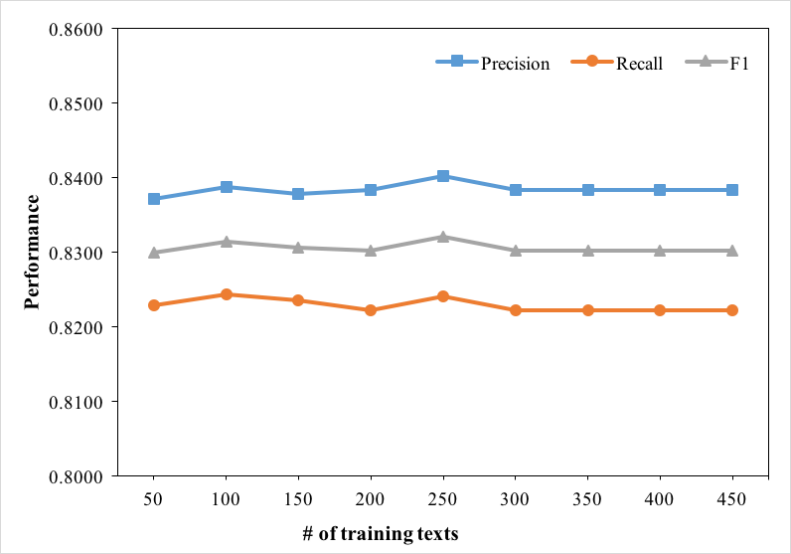
Performance changes using the method with different sizes of training dataset.

**Figure 8:**
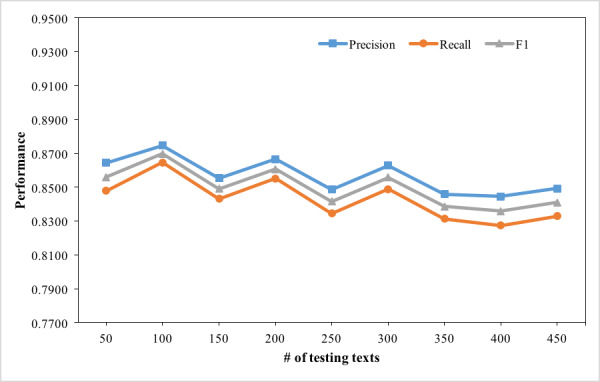
Experiment result of the stability test.

Patterns and temporal features were generated from the training dataset, which indicated that the scale of training dataset might influence the effectiveness of extraction and normalization. We used different sizes of the training dataset to discover how the scale of training dataset affected the performance. In the experiment, the number of EMRs in the testing dataset remained the same (450), while the number of training datasets increased from 50 to 450. The experiment result is presented by a line chart in Figure 7. The experiment result showed that the scale of training dataset had slight influence on the performance of the approach. When the number of EMRs from the training dataset reached 300, the effectiveness of the training dataset tended to be stable. Through analyzing the dataset and experiment result, we found the types, relative positions, and contexts of TEs in each discharge summary were mostly similar. With the number of EMRs in training dataset increasing, the number of learned patterns and generated temporal feature vectors increased. However, the influence of the same patterns and vectors on TNorm was steady and independent of their quantity. In addition, we also tested the stability of TNorm using different sizes of testing datasets (50 to 450) but kept the training dataset the same (450). The result, as Figure 8 shows, illustrates that TNorm reached a comparative stable performance when the number of EMRs was larger than 350. In this experiment, when the testing dataset was increased to 450 records, TNorm achieved a precision of 0.8491, a recall of 0.8328, and an F1 score of 0.8409. With the testing dataset scale increasing, the performance of the F1 score changed slightly.

**Table 4:** Detailed experiment result of the top 10 classification algorithms.

Classification algorithm	Macro-average precision	Macro-average recall	Macro-average F1
Multiclass classifier	0.9553	0.9420	0.9485
Logistic	0.9558	0.9425	0.9488
Simple logistic	0.9560	0.9423	0.9490
Iterative classifier optimizer	0.9493	0.9525	0.9510
Logit boost	0.9493	0.9525	0.9510
Decision table	0.9493	0.9538	0.9513
JRip	0.9523	0.9518	0.9523
K-nearest neighbor (k=1)	0.9518	0.9613	0.9563
Logistic model trees	0.9545	0.9598	0.9570
Randomizable filtered classifier	0.9535	0.9613	0.9573

**Table 5:** Evaluation result of the efficiency of the learned patterns in TNorm.

Strategy		Accuracy
**Randomizable filtered classifier**		
	rule	0.8587
	rule+pattern	0.8654
**Logistic model trees**		
	rule	0.8587
	rule+pattern	0.8654
**K-nearest neighbor (k=1)**		
	rule	0.8587
	rule+pattern	0.8654
**JRip**		
	rule	0.8586
	rule+pattern	0.8653
**Decision table**		
	rule	0.8586
	rule+pattern	0.8653

## Discussion

### Principal Findings

In the experiment of comparing classification algorithms for temporal type prediction, we used the macro-average F1-measure as the ultimate evaluation metric and finally selected RFC as the best to be integrated in TNorm. Throughout the experiment, TNorm performed well on the combination task of TE extraction, temporal type prediction, and temporal normalization. However, a few errors still occurred in each process of applying TNorm.

All error cases from TNorm were analyzed and classified to three types. The first was errors caused by wrong representations or typos in original narrative texts. For instance, “*出院日期*” (discharge date) followed by the temporal information of discharge date was normally mentioned in every clinical discharge summary. However, in some special discharge summaries, the date was incorrectly written as “*出院日期:出院日期*” (discharge date: discharge date), which caused the problem of lacking specific discharge date information. As a result, in this wrongly representative text, the TE that required the discharge date as reference time could not be normalized correctly. In addition, some clinical texts contained a series of TEs (eg, the text “*门诊 星期一 星期二 星期三 星期四 星期五 上午*” [Outpatient Monday Tuesday Wednesday Thursday Friday AM]) without represented any specific time, causing difficulty in normalization.

The second error type was caused by machine learning algorithms for temporal type prediction. Since the rules of temporal normalization were associated with temporal types, TEs with wrong temporal types might be matched with inappropriate rules, causing negative effects in the normalization. For example, in the text “*肿物增大2月*” example, the text “*2月*” was classified as the type “DURATION,” but the classification algorithm predicted it as “DATE.” The incorrect type label resulted in false temporal value (eg, correct normalization result should be “*肿物增大*<TIMEX3 tid=“t7” Type=“DURATION” Value=“P2M”> *2月*</TIMEX3>” while the result generated by TNorm was “*肿物增大*<TIMEX3 tid=“t7” Type=“DATE” Value=“2014-2” anchorTimeID=“t3”> *2月*</TIMEX3>”).

The third error type was caused by automatically generated patterns. Although the learned patterns could improve the precision of TNorm, they might cause matching mistakes in special cases. For example, the pattern “ 于我” matched matched the text “*6天前于我院行双乳B超*,” thus a temporal value “2014-10-23,” which was the same as the normalized value of its reference time, was computed. However, the correct standardized value of the TE “*6天前*” was “2014-10-17.”

### Limitations

There was a limitation of the proposed method. The TNorm consisted of sequential functions including (1) TE extraction, (2) temporal type prediction, and (3) TE normalization. Since time expressions were processed step by step in a sequence order, any errors generated from a step in the process might have negative effects in the next step. To reduce or eliminate this kind of effect, we will try to explore a joint model that conducts the three tasks of extraction, predication, and normalization simultaneously in the future.

### Conclusions

This paper proposed a method, TNorm, for automatically extracting and normalizing TEs from Chinese narrative clinical texts. TNorm was composed of alternative machine learning methods, a pattern learning method, and a set of heuristic rules. Several experiments based on 1459 Chinese clinical texts from a 3A hospital in mainland China were conducted to evaluate the performance of classification algorithms, effectiveness of pattern learning, and stability of TNorm, respectively. Results demonstrated that TNorm was reliable and stable for TE normalization of Chinese EMR records.

## Abbreviations

CRF: conditional random field
EMR: electronic medical record
ISO: International Organization for Standardization
KNN: k-nearest neighbor
NLP: natural language processing
NLTK: Natural Language Toolkit
RFC: randomizable filtered classifier
RI-TIMEX: relative and incomplete temporal expression
TE: temporal expression
tid: Timex ID number
Weka: Waikato Environment for Knowledge Analysis
